# Experiences and observations from a care point for displaced Ukrainians: a community case study in Antwerp, Belgium

**DOI:** 10.3389/fpubh.2024.1349364

**Published:** 2024-06-26

**Authors:** Stefan Morreel, Veronique Verhoeven, Hilde Bastiaens, Katrien Monten, Josefien van Olmen

**Affiliations:** ^1^Department of Family Medicine and Population Health, University of Antwerp, Antwerp, Belgium; ^2^Department of Health, Antwerp, Belgium

**Keywords:** Belgium, Ukraine, health systems, refugees, healthcare organization, primary care, nursing

## Abstract

**Background:**

A total of 7,307 Ukrainian refugees moved to Antwerp, Belgium, during the study period (01 April 2022 to 31 December 2022). The city’s administration set up three care centers where these people were introduced to the Belgian primary care system, a medical file was created, and acute/preventive/chronic care was delivered. This community case study analyzes the organization and contents of care and reflects upon its meaning for the mainstream healthcare system.

**Methods:**

This is an observational study using routine electronic medical record data to measure the uptake of care. For a sample of 200 subjects, a retrospective chart review was conducted.

**Participants:**

All refugees with a medical file at one of the three participating care centers were included.

**Main outcomes:**

For the observational study, 2,261 patients were reached (30% of the potential users), and 6,450 contacts were studied. The nurses (including midwives) conducted 4,929 out of 6,450 (76%) of all consultations, while the general practitioners (GPs) conducted 1,521 out of 6,450 (24%). Of the nurse consultations, 955 (19%) were followed by another nurse consultation and 866 (18%) by a GP consultation. In the structured case reviews, most contacts were concerned with acute problems (609 out of 1,074, 57%). The most prevalent reasons for encounters and diagnoses were typical primary care issues. The nurses were able to manage half of the cases independently (327, 55%), referred 37% (217) of cases to the GP, and consulted a GP (live, by telephone, or a dedicated app) for 8% (48) of cases. GPs mostly prescribed drugs, referred to a medical specialist, and advised over-the-counter drugs, while nurses more often advised over-the-counter drugs (mostly paracetamol, nose sprays, and anti-inflammatory drugs), provided non-medical advice, or ordered laboratory tests.

**Discussion:**

The medical care points delivered mostly typical acute primary care in this first phase, with a key role for nurses. The care points did not sufficiently take up chronic diseases and mental health problems. These results will inform policymakers on the use of primary care centers for newly arriving patients in times of a large influx. A nurse-first model seems feasible and efficient, but evaluation of safety and quality of care is needed. Once the acute phase of this crisis fades away, questions about the comprehensiveness, continuity, and integration of care for migrants remain relevant.

## 1 Introduction

International crises, such as war, persecution, and natural disasters, force people to move from their home environment. Other countries need to prepare themselves to welcome refugees and provide shelter. This means that destination countries need to think about how to address the needs of newcomers for health and wellbeing, which includes housing, education, employment, health, social inclusion and care, and other social services (1). The influx of forced migrants tends to occur in waves that are partially unpredictable. Since destination countries tend to scale down their refugee support in times of low arrivals, the rise is—repeatedly—perceived as an acute crisis for which administrative and support systems are insufficiently prepared (2). The response of government actors, therefore, bears an element of pressure that requires an acute investment of time and money. At the same time, it can unleash new energy and offer out-of-the-box solutions. In this way, refugee crises resemble other crises that put stress on healthcare systems, such as COVID-19.

The organization of healthcare for forced migrants in Europe has gained increasing attention in recent decades, partly because of the human catastrophes during and after their journey depicted in the media and partly because of the framing of migrant health as a health security issue with an impact on the general population (3). Primary healthcare is already under pressure, and an influx of more people, with sometimes complex problems complicated by a language barrier, poses new challenges that can compromise the quality of healthcare for the general population. The emergence of infectious diseases due to overcrowding can, for instance, pose a risk to the general population. Barriers to access to healthcare are highly prevalent in migrant populations and can lead to underuse and misuse of healthcare with potentially increased healthcare costs. The barriers to access and quality of care at the operational level have been well described from both user (4) and provider (5) perspectives. These include language and communication barriers, differences in sociocultural norms and illness perceptions, low health literacy and the failure to understand or navigate new healthcare systems, perceived discrimination and low trust in the system, and precarious circumstances interfering with health and healthcare access, such as social deprivation, trauma, and lack of insurance coverage (6). Despite these studies identifying clear gaps, there is little knowledge of system-level interventions to improve the performance of healthcare services that would benefit both forced migrants and host societies (7). For instance, how to organize care that is accessible and addresses the actual needs of forced migrants, which data to collect to optimize patient care, and who is best placed to provide care?

The Belgian healthcare system is organized into primary, secondary, and tertiary care, with direct access for patients at all levels. It is mainly organized as a fee-for-service system. This system covers almost the entire population for a wide range of health services. Residents must be affiliated with a sickness fund; contributions are proportional to income. Belgium is among the top 10 spenders on health across EU countries, with up to 10.7% of its GDP in 2019 (8). The increasing number of people with chronic diseases and staff shortages led to the overburdening of the primary care system in Belgium, especially in larger cities, resulting in patient stops and waiting lists (9).

According to European Union legislation (among others, directive 2013/33/EU), Belgium needs to fulfill the basic needs of asylum seekers during their asylum procedure (10). Most asylum seekers live in asylum centers operated by the government or NGOs. These centers have a frontline medical service with nurses and doctors. A minority lives with friends or relatives within the community and relies on the regular healthcare system. A stakeholder consultation identified a number of problems in medical care for people in the asylum process; at the macro level: a lack of coordination, regional differences, a lack of monitoring utilization and costs of care, a lack of understanding about care expenditures, and a lack of administrative support; at the meso level: unclear administrative system for healthcare providers, differences in care according to the place of residence, lack of qualified healthcare providers available, large turnover and overcrowding of health services, unclear agreements on collaboration, tension over data confidentiality, and lack of a medical information system; and at the micro level: inequities in access, treatment, and health outcomes (11).

The aim of this community case study is to analyze a local health initiative set up to address the needs of a sudden influx of forced migrants (from Ukraine). It addresses several subjects from a health service perspective: organization of care delivery; process outcomes in terms of delivery of care and population reached; and content of care. The results of this study are relevant for the future care of the target population and might inspire healthcare organizations for other target populations and crisis contexts.

The studied local health initiative originates from a government initiative to tackle the medical needs of an (at that time) expected large influx of Ukrainian refugees. The Belgian federal government (responsible for most of the crisis management and the healthcare system) asked the Flemish regional government (responsible for prevention and the organization of primary care) to set up local initiatives to address the health needs of these refugees. These local initiatives consisted of care points (“Zorgpunten” in Dutch), which had to be set up by local primary care zones. These zones are a collaboration of the local government, healthcare workers, and social welfare organizations to facilitate healthcare professionals to deliver person-centered, accessible, and high-quality primary care (12). In the city of Antwerp (part of Flanders), four primary care zones started a combined initiative, which will further be called the “care points.” These Antwerp care points emerged from a similar local initiative to combat COVID-19 (13). By coincidence, the end of the COVID-19 crisis coincided with the arrival of the first Ukrainian refugees. The primary care zones had exceptional liberty in organizing these care points. They were financed *per capita* in contrast to most of the Belgian healthcare system.

In this study, we describe the initiative and report the results of a retrospective observational study using routine data from electronic files to assess the process and content of care. For a sample of 200 subjects, an additional retrospective chart review was conducted to complement and deepen this data (14).

For the observational study, all recorded contacts at the care points during the study period (31 March 2022–31 December 2022) were assessed for eligibility (*N* = 8,346). Contacts for non-Ukrainian citizens, no-shows, questions answered by the administrative staff, telephone consultations, and consultations beyond the study period were excluded. Data were extracted from the care point’s electronic patient files (Mediris Multi®) by a staff member of the care points. The data were registered by GPs and nurses during their consultations; they were not given any research instructions. Psychologists did not use this system; no data was available on their delivered care. These electronic patient files contain free text fields (consultation report and conclusion) and structured fields (patient sex, time of consultation, caregiver, and type of contact). The structured data fields, namely patient names, healthcare professional names, and healthcare professional registration numbers, were pseudonymized using the irreversible SHA 256 algorithm by the staff member. The patient’s date of birth was replaced by age after data cleaning. For most patients, a structured assessment of their prevention needs primarily focused on tuberculosis screening, and vaccinations were conducted and registered in structured data fields, mostly during their initial encounter (intake). The authors also intended to study these preventive assessments, but the data were unsuitable for analysis due to registration flaws. While these contacts were included in the overall number of consultations, specific prevention outcomes were not analyzed.

For the retrospective chart review, a care point administrative staff member extracted a random sample of 200 patient files. The software ordered all 2,261 files alphabetically, and the staff member picked every 11th file. This staff member replaced the names of the professionals by their profession (MD, nurse, midwife, or administrative staff), erased the name of the patient, and replaced the birthdate with the birth year. Author SM coded the first 10 files, after which all authors except KM drafted a codebook. This codebook was evaluated on 10 more files jointly coded by the same authors. This codebook (in Dutch) is available upon request. A single author coded each file. Although most consultations involved one problem (one subcontact), some consultations consisted of several subcontacts concerning distinct clinical problems (e.g., a patient came to discuss arterial hypertension and a rash). Data fields included the timestamp of the consultation, discipline of the caregiver, type of contact (new problem, follow-up, intake, prevention only, and technical procedure only), chronicity (chronic or acute), the reason for encounter (code according to the International classification of Primary Care version 2, ICPC-2), diagnosis (ICPC-2), and actions. The reason for the encounter and the diagnosis were not strictly restricted to medical subjects; procedures or administrative tasks could also be registered. In the case of a nurse consultation, the variable interprofessional workflow (nurse works independently, nurse refers to MD, or nurse asks MD advice) was assessed as well. The preventive assessment contacts were excluded from the retrospective chart review unless they also comprised non-preventive data.

Raw data for the retrospective chart review were collected using a structured spreadsheet in Microsoft Excel®. All data were analyzed using JMP Pro® version 17. Descriptive statistics and the Pearson chi-square tests were used.

## 2 Context

### 2.1 The influx of Ukrainian refugees

From the start of the war between Russia and Ukraine in February 2021 until the end of 2023, 7.9 million refugees had fled Ukraine (15). More than 60% of these refugees suffer from substantial or severe psychological distress, which poses challenges, especially in combination with the health risks of their migration (16, 17). Most of them fled to neighboring countries, such as Poland. Unlike most other refugees, Ukrainians are treated as EU citizens in the entire EU, giving them the right to temporary protection. These rights include social rights and access to health insurance (18, 19). EU healthcare systems are trying to adjust to the influx of people from Ukraine and the related healthcare needs, such as physical and mental trauma related to the war (20). Data about the health and healthcare provided to Ukrainian refugees are so far scarce, hampering response and future planning (21).

### 2.2 Community context

In Belgium, 65,000 Ukrainian refugees arrived by the end of 2022. The majority (62%) were female and 34% were minors. Only 16% demanded shelter, and the other 84% found shelter themselves (mostly friends or relatives) (15). Ukrainian refugees needed to register themselves to obtain social rights, including health insurance.

The current community case is situated in Antwerp, a city with a majority of its 532,000 people having roots outside of Belgium (22). In 2022, 7,307 Ukrainians registered themselves at the municipality (59% female, average age 29), although the actual figure is probably higher. No reliable data on the departing number of Ukrainians (either traveling further or returning to their homes) were available.

## 3 Key programmatic elements

### 3.1 Organization of care

The routine primary care system was already being overstretched, so the city of Antwerp decided to organize medical care for this large new population in a separate system. Three care points with complementary functions were set up across the city. The first care point was situated in an acute refugee shelter set up in March 2022 to organize the first welcome and a medical/social intake of new arrivals. This center was closed on 1 November 2022 because the influx became more gradual and predictable. Most of the refugees found a home within the community, either with or without support from the city’s administration. For them, a second ambulatory care point was set up on 31 March 2022. The large influx of people pushed the city to create additional accommodation facilities in the form of an emergency container village at the edge of the city for approximately 700 people. This location hosted the third care point set up on the same date.

The care points were staffed by nurses hired by the city of Antwerp and by independent GPs who worked a limited number of hours per week in addition to their routine work. GPs were remunerated on an hourly basis. The person in charge of the care points was a senior nurse with experience in refugee care. Most of the nurses hired also had experience working in a refugee setting. Apart from providing care, they were actively involved in on-the-job peer training of other staff in the care points. These care points were free of charge to the patients. Nurses were the first point of contact. Patients could access a GP only after a nurse had done the first intake and decided this was necessary. Some chronic patients were actively transferred to regular primary or secondary care, but no data are available on these transfers. An appointment with a psychologist alone or in a group was possible upon the request of the patient. When booking a consultation, patients were always able to simultaneously book a Ukrainian or Russian interpreter. These interpreters were volunteers trained on the job.

The city aimed for optimal access to both the preventive and curative care services provided by the care points. At the moment of registration at the city’s admin and social services, people were asked to sign an informed consent form (available in Dutch, Ukrainian, and Russian). During registration, a Ukrainian interpreter and a Russian interpreter were present. In the informed consent form, refugees gave permission to be contacted for preventive care and a medical intake. Complementarily, the city distributed information on the care points through leaflets (in Dutch and Ukrainian), text messages (in Dutch and Ukrainian), a website (Dutch only), and explanations during civic integration courses. Care points would then contact people to offer an intake that offered preventive screening and care and curative care if necessary. Primary care professionals were also informed so that they could refer people.

### 3.2 Process outcomes and content of care: observational study

By the end of this study, 6,722 out of 7,307 registered refugees (91%) signed the informed consent, and only two explicitly refused. The remaining 583 did not sign, most likely because the administration forgot to ask them or the signature got lost.

From 31 March 2022 to 31 December 2022, 2,261 patients were registered at the care points (30% of all registered refugees and 34% of those who signed the informed consent). The total number of healthcare contacts registered in that period was 8,346. Contacts for non-Ukrainian citizens (*N* = 24), no-shows (*N* = 117), questions answered by the administrative staff (1,152), telephone consultations (*N* = 2), and consultations beyond the study period (*N* = 558) were excluded, leading to a total of 6,450 studied contacts. The mean number of contacts was 2.87 (standard deviation 2.71, median 2). The majority was female (59%). The mean age of the patients was 27 years (male patients 24 years and female patients 30 years). The nurses conducted 4,863 out of 6,450 (75%) of all consultations, while the GPs 1,521 out of 6,450 (24%) and midwives 66 out of 6,450 (1%). Because of the small number of midwife consultations, consultations by midwives and nurses were taken together in the analyses. Reporting them separately could compromise the anonymity of this report.

Sex did not influence the odds of getting a nurse consultation (chi-square *p*-value = 0.59). Older patients less frequently saw a nurse (chi-square *p*-value < 0.01): children below 12 years of age saw a nurse for 81% of their consultations, whereas for patients aged 65–85 years, this proportion was 65%.

Thirty-two GPs performed between 2 and 191 consultations (mean 48, standard deviation 57). Six GPs conducted more than 100 consultations each. A total of 38 nurses executed between 1 and 806 consultations (mean 130, standard deviation 197), and 12 nurses conducted more than 100 consultations each.

To analyze the interprofessional patient flows, we studied those consultations where the same patient was seen twice within 7 days. Of the 4,929 nurse consultations, 955 (19%) were followed by another nurse consultation and 866 (18%) by a GP consultation. Of the 1,521 GP consultations, 281 (18%) were followed by a nurse consultation and 136 (9%) by another GP consultation. Children below 12 years of age had 1,586 nurse consultations, of which 281 (18%) were followed by a nurse consultation and 214 (13%) by a GP. On the contrary, for patients 65–85 years of age, 246 nurse consultations were followed by 71 (29%) nurse consultations and 77 (31%) GP consultations.

### 3.3 Process outcomes and content of care: retrospective chart review

Two hundred patient files were selected randomly. A total of 991 consultations were coded concerning 193 different patients. In total, 7 of the 200 patient files were either empty or duplicates. The subpopulation of 193 patients was demographically similar to the overall population of 2,261 patients, with a mean age of 28 years and 56% female patients. In 69 consultations, people mentioned numerous unrelated complaints, which were coded as subcontacts, leading to a total of 1,073 contacts. Each author except KM coded at least 200 contacts. Most of the contacts (624/992 or 63%) were conducted by nurses, 356 out of 992 (36%) by MDs, and 3 out of 992 (0%, further analyzed in the group of nurses) by midwives. For nine contacts, the caregiver was unknown. The proportion of nurse contacts (63%) was lower in the retrospective chart review as compared to the observational study population (75%), most likely because preventive consultations (which were all done by the nurses) were excluded from this analysis.

Most consultations were concerned with acute problems (609/1,074, 57%). Half of the contacts (546, 51%) were concerned with a new complaint, 388 (36%) were follow-up, and the remaining 139 (13%) were concerned with an intake consultation, non-routine prevention actions, and technical procedures, such as injections. See Table 1 for an overview of the most common reasons for encounter. Most contacts were concerned with respiratory complaints (193, 18% with coughing as the most frequent complaint), procedures and administration (165, 15% with request for prescriptions as the most frequent subject), general and unspecified complaints (134, 12% with fever as the most frequent subject), and musculoskeletal complaints (108, 10% with back pain as the most frequent subject).

**Table 1:** Reasons for encounter at the care points split into ICPC-2 chapters.

Reason for encounter (ICPC-2 Chapter)	*N*	Column %
Respiratory	193	18%
Procedures and administration	165	15%
General and unspecified	134	12%
Musculoskeletal	108	10%
Skin	77	7%
Digestive	59	5%
Cardiovascular	54	5%
Missing	49	5%
Female genital	41	4%
Neurological	35	3%
Ear	32	3%
Endocrine/metabolic and nutritional	31	3%
Psychological	29	3%
Urological	21	2%
Eye	18	2%
Pregnancy, childbearing, family planning	11	1%
Male Genital	8	1%
Blood, blood forming organs, and immune mechanism	6	1%
Social problems	2	0%
All	1,073	100%

Source: structured case review. ICPC-2, International classification of Primary Care version 2.

We compared the final coding of evaluation and actions of GPs and nurses, illustrating the difference in the type of actions of both professions. See Table 2 for an overview of the GP’s most common diagnoses, which were logically similar to the reasons for the encounter, and the same applies to the nurse’s diagnoses (see Table 3). In total, 819 actions were coded; multiple actions were possible for a single subcontact. The management of the cases was different for nurses and GPs. GPs mostly prescribed drugs, referred to a medical specialist, and advised over-the-counter drugs, while nurses more often advised over-the-counter drugs (mostly paracetamol, nose sprays, and anti-inflammatory drugs), provided non-medical advice (e.g., “Rinse your nose and take a spoon of honey”), or ordered laboratory tests (mostly point of care COVID tests). For 385 out of 1,073 contacts (36%), no information on actions was recorded. See Tables 4, 5 for more details.

**Table 2:** GP diagnoses at the care points split into ICPC-2 chapters.

Diagnosis (ICPC-2 Chapter)	*N*	Column %
Respiratory	59	15%
General and unspecified	53	13%
Musculoskeletal	53	13%
Procedures and administration	51	13%
Skin	28	7%
Cardiovascular	24	6%
Female genital	21	5%
Digestive	18	5%
Neurological	16	4%
Endocrine/metabolic and nutritional	15	4%
Psychological	14	4%
Ear	11	3%
Urological	11	3%
Missing	8	2%
Eye	8	2%
Male genital	4	1%
Blood, blood forming organs, and immune mechanism	3	1%
Pregnancy, childbearing, family planning	2	1%
All	399	100%

Source: structured case review. ICPC-2, International classification of Primary Care version 2.

**Table 3:** Nurse diagnoses at the care points split into ICPC-2 chapters.

Diagnosis (ICPC-2 Chapter)	*N*	Column %
Respiratory	134	20%
Procedures and administration	113	17%
General and unspecified	80	12%
Musculoskeletal	55	8%
Skin	48	7%
Missing	41	6%
Digestive	41	6%
Cardiovascular	30	5%
Ear	21	3%
Neurological	19	3%
Female genital	18	3%
Endocrine/metabolic and nutritional	15	2%
Psychological	14	2%
Eye	10	2%
Urological	9	1%
Pregnancy, childbearing, family planning	8	1%
Male genital	4	1%
Blood, blood forming organs, and immune mechanism	3	0%
Social problems	2	0%
All	665	100%

Source: structured case review. ICPC-2, International classification of Primary Care version 2.

**Table 4:** GP management actions at the care points.

Management actions	*N*	Column %
Drug prescription	89	21%
Referral to a specialist	63	15%
Advice on over-the-counter drugs	60	14%
Renewal of a prescription drug	36	8%
Reassurance	31	7%
Non-medical advice	29	7%
Laboratory testing	28	7%
Adjustment of a prescription drug	20	5%
Medical imaging	18	4%
Physiotherapy	18	4%
Other intervention	14	3%
Advice to continue over-the-counter drugs	9	2%
Other technical examination	6	1%
Referral to dentist	4	1%
Adjustment of over-the-counter drugs	1	0%
All	426	100%

Source: structured case review.

**Table 5:** Nurse management actions at the care points.

Management actions	*N*	Column %
Advice on over-the-counter drugs	121	31%
Non-medical advice	68	17%
Laboratory testing	43	11%
Referral to a specialist	38	10%
Reassurance	31	8%
Asked the GP to prescribe a drug	29	7%
Asked the GP to renew a prescription drug	20	5%
Other intervention	14	4%
Vaccination	8	2%
Advice to continue over-the-counter drugs	6	2%
Referral to dentist	4	1%
Medical imaging	3	1%
Other technical examination	3	1%
Adjustment of over-the-counter drugs	2	1%
Physiotherapy	2	1%
Adjustment of a prescription drug	1	0%
All	393	100%

Source: structured case review.

For 592 out of the 624 nurse consultations (95%), information on the interprofessional workflow was available. The nurses were able to manage half of the cases alone (327, 55%), referred to the GP in 37% (*N* = 217), and consulted a GP (live, by telephone, or a dedicated app) for 8% (48).

## 4 Discussion

In this community case report, we described how local authorities organized medical services for a group of Ukrainian refugees in a Belgian city through a system of dedicated care points outside of the mainstream health system. The observations provide opportunities to reflect upon the process outcomes coverage and utilization, as well as the content of care, and to reflect upon the lessons both for the organization of care for newly arriving migrants and the mainstream healthcare system.

Despite repeated information campaigns, the care points only reached 30% of the population, leaving room for improvement. For example, 8% of the registered refugees did not sign nor refuse informed consent, and the administrative process should be reinforced here. Those who made use of the care points had an average of 2.87 consultations per person in 9 months, which seems comparable to the United Nations Refugee Agency’s indicative statistics of emergency standard health facility utilization of 1–4 consultations per person per year. No information is available about if and where the other 70% of the Ukrainian refugees sought preventive or curative care.

The content of care mostly pertained to typical acute primary care illnesses, which is in line with the major population being children and young women and with first-phase care in other settings (23). While Ukrainian refugees in Germany reported high psychological stress and mental health problems, the Antwerp figures do not show a large uptake of mental issues by nurses or GPs at the care points in the first 9 months (16). It seems unlikely for Ukrainian refugees in Belgium to have less psychological stress; most likely, the nurses and GPs at the care points did not pick up these problems because of several barriers: staff was not specifically trained, psychologists were only available upon request, and no systematic screening was set up. In addition, we also found that few consultations included chronic care. This is surprising as the war has severely disrupted chronic care services in Ukraine. While 30% of households have a person with at least one chronic condition, many have not been able to access care since the war (24). Before the war, the Ukrainians were used to a health system with a package of health services for the population, including chronic care and yearly check-ups, albeit with relatively high co-payment (24, 25). This study does not reveal a reason for this finding: is this young refugee population in relatively good health, or did the care points not address or detect chronic problems well? It seems that more complex problems, such as mental health or chronic problems, were not being revealed. Potential explanations can be patient-related (for instance, language barrier, not having trust in the healthcare provider), provider-related (not feeling capable of addressing such problems), interpreter-related, or organization-related (lack of time). These findings on the contents of care resemble studies on out-of-hours primary care, showing that people present with primordially minor urgent problems (26). Further research is necessary on this subject.

Local initiators and city authorities decided upon the organization of care. Higher-level governments are supported through financial reimbursement and regulatory flexibility. The vast majority of the work was done by nurses, who were the gatekeepers for GP consultations. The nurses performed more than half of the consultations autonomously. Especially in children, they handled the vast majority of the work and the follow-up consultations. This community case study shows that it is feasible to set up a nurse-led care provision supported by GPs for acute, common disorders and standardized preventive care for a specific population and their needs. This is also a model used in the regular Belgian system for asylum seekers (27). These results should be regarded as a broader trend toward the integration of nurses in primary care (28). Nurse-led care is still relatively underdeveloped in primary care in Belgium. Practices are mainly staffed with GPs, and if nurses are employed, they often do mainly technical acts or administrative support. A constraint in adapting this is the fee-for-service financing model in which GPs are not paid for time spent outside the direct patient contact, for instance, liaising with nurses or for care delivered by nurses. This barrier was not present in this community case.

The studied care points are in line with new organizational care models that take into account interprofessional collaboration and task delegation in the context of an aging population and increased pressure on primary care (29, 30). This care point case study provides a setting with experienced nurses in a more autonomous role compared to the current mainstream primary care practices in Belgium. Belgium has a relative shortage of GPs due to the aging of the GPs and low attrition of young GPs, so installing more nurses in regular primary care might be productive (29, 30). The study period was too short, and the sample was too small to allow for any conclusions regarding the safety of the studied nurse-first care model. In addition, the current shortage and lack of suitable training for such a cadre of nurses renders it challenging to apply this model to the regular Belgian primary care system at this moment. When this study was conducted, there were no formal guidelines for nurses in primary care in Belgium, so it was not possible to determine whether the care responses of the nurses were concordant with the standard of care.

Another novelty regarding nurse-led care in primary care is the way nurses register medical diagnoses. Although they slightly differ from the medical diagnoses recorded by GPs, it is compelling that nurses register medical diagnoses even without involving a GP, such as an acute middle ear infection. Nurses did not make use of nursing diagnoses (e.g., acute pain in the ear), which are more problem-focused (31). This finding might be explained by two factors. First, nurses were not specifically trained to make diagnoses. Second, the software used was designed for doctors.

While the Ukraine war lingers on and new crises appear, the necessity to think about sustainability becomes urgent. The continuation of the current model raises challenges in terms of comprehensiveness and equity. The current model is not designed to offer chronic and mental care, while these care needs are expected to increase over time. While the high levels of solidarity of European populations with the Ukrainian population contributed to a differentiated approach to Ukraine refugees in the initial phase of the war, this became less obvious over time (32). For instance, other people in vulnerable situations (refugees from other countries, homeless people, illegal substance users, etc.) do not have equal access to a specialized care point and do not have the same access to European health insurance either. Local initiatives such as the Antwerp care points can be complementary to other existing structures for refugee services, but coordination and alignment of resources and processes are necessary. Currently, a long-term follow-up system is necessary as it is not feasible to transfer the entire workload to the regular primary care system, nor is it desirable to continue the current way of working.

In addition, policymakers need to develop a strategy for integrating migrants into mainstream health service systems in a way that prevents overburdening. Examples from other countries, such as Quebec, where the integration process is streamlined through a navigator person for migrants, additional training for GPs, and translation services, provide clues for success (33).

We conclude that local care points were useful for the medical welcome of a sudden influx of refugees. Nurses worked efficiently and only deployed GPs when necessary. Many questions remain concerning the generalizability of these results, the safety of the tested care model, and the continuity of care after the acute influx fades away.

## 5 Acknowledgment of conceptual and methodological constraints

This study has the typical limitations of a longitudinal retrospective study without a control group. Comparison of the results to regular Belgian primary care in terms of reasons for encounter, diagnoses, consultation uptake, and case management was not possible. Although planned, no reliable data concerning the delivery of preventive care were available. No data on the care delivered by the psychologists were available for this study. The authors were only able to assess the interdisciplinarity follow-up of nurse consultations; no reliable data concerning the interdisciplinary follow-up of physician consultations was available. The authors only looked at the registration in the electronic medical records. This does not present a representative overview of the health problems of the Ukrainian refugee population. The healthcare workers had no tools to specifically register mental health or migration stress issues.

The patient sample for the retrospective chart review was randomly drawn, but apart from age and sex, the representativeness as compared to the overall population was not studied. Similarly, we do not know whether the population reached by the care points was similar to the unreached population. As reaching patients was not random, a selection bias is likely. A feasibility sample of 200 patients was chosen; no formal sample size calculation was possible because of the lack of previous studies.

The codebook was jointly created by the authors but afterward, every case was only judged once because of a lack of funding. The intra- and interrater variability has not been tested. The codebook was based upon variables found in the files; selective registration of certain variables is possible as the patient files were destined for clinical use, not research.

The authors only studied quantitative data. A companion qualitative study addressing the barriers and facilitators of the studied care model is necessary. Such a study should involve focus groups and in-depth interviews with patients and refugees who are not reached by the care points, stakeholders, policymakers, and healthcare professionals.

## Data availability statement

The datasets presented in this article are not readily available because the studied data is not sharable because of a lack of individual consent. The authors are however allowed to share the raw data for specific variables upon reasonable request and if in line with ethical regulations. The raw data of the structured case reviews can never be shared. Requests to access the datasets should be directed to stefan.morreel@uantwerpen.be.

## Ethics statement

The studies involving humans were approved by Antwerp University Hospital Institutional Review Board (project ID 3952) on 14/11/2022. The studies were conducted in accordance with the local legislation and institutional requirements. The ethics committee/institutional review board waived the requirement of written informed consent for participation from the participants or the participants’ legal guardians/next of kin because pseudonymized files were used, individual consent was not possible.

## Author contributions

SM: Conceptualization, Data curation, Formal analysis, Investigation, Methodology, Project administration, Resources, Software, Validation, Writing – original draft, Writing – review & editing. VV: Conceptualization, Investigation, Methodology, Writing – review & editing. HB: Conceptualization, Investigation, Methodology, Writing – review & editing. KM: Conceptualization, Writing – review & editing. JO: Conceptualization, Investigation, Methodology, Writing – original draft, Writing – review & editing.
